# Isolation and molecular identification of pathogens causing sea turtle egg fusariosis in key nesting beaches in Costa Rica

**DOI:** 10.1371/journal.pone.0333280

**Published:** 2025-09-25

**Authors:** Keilor E. Cordero-Umaña, Ruth Hernando-Martínez, María Martínez-Ríos, Jaime Restrepo, Roldán A. Valverde, Laura Martín-Torrijos, Pilar Santidrián Tomillo, Javier Diéguez-Uribeondo

**Affiliations:** 1 Department of Mycology, Real Jardín Botánico (RJB), CSIC, Madrid, Spain; 2 The Leatherback Trust, Goldring-Gund Marine Biology Station, Playa Grande, Costa Rica; 3 Centre for Biodiversity and Conservation Science, The University of Queensland, St. Lucia, Queensland, Australia; 4 Sea Turtle Conservancy, Gainesville, Florida, United States of America; 5 The University of Texas Rio Grande Valley, Brownsville, Texas, United States of America; 6 Centre Oceanogràfic de Illes Balears, Instituto Español de Oceanografía (COB-IEO), CSIC, Palma de Mallorca, Spain; University of Basrah, IRAQ

## Abstract

The global rise of fungal pathogens presents an emerging threat to biodiversity, with significant risks to species such as endangered sea turtles. The fungal disease known as sea turtle egg fusariosis (STEF) is associated with high embryo mortality rates and represents a substantial conservation challenge. This disease is caused by two fungal species, namely *Fusarium falciforme* (*Ff)* and *Fusarium keratoplasticum* (*Fk*), and their identification is essential for guiding future efforts to address potential fungal infections, particularly on important nesting beaches such as those in Costa Rica. In this study, we conducted fungal isolations from sea turtle eggshells and nest sand at four key nesting beaches along the Pacific and Caribbean coasts of Costa Rica to evaluate the presence of STEF-causing species. For accurate identification, we employed a multilocus sequence typing (MLST) approach, analyzing three genetic loci. We obtained 147 axenic cultures, of which 32% belonged to the STEF-causing species *Ff* (n = 32) and *Fk* (n = 15). *Fusarium falciforme* was found across all study locations on both coasts of Costa Rica, whereas *Fk* was only detected at one beach on the Caribbean coast. This study represents the first survey to accurately identify STEF-causing species in Costa Rica, revealing a widespread presence on the main nesting beaches. Currently, STEF is not severely affecting sea turtles in Costa Rica; however, various factors, such as changes in the nesting beach environment and sand composition, could increase the incidence and severity of the disease, posing a threatening risk to embryonic development. Therefore, a better understanding of the presence and distribution of these pathogens is critical for preventing the development of this emerging disease.

## Introduction

Numerous taxa worldwide have been affected by highly virulent emerging fungal diseases [1,2]. Some of these diseases have captured the attention of the scientific community due to their high mortality rates, particularly among threatened species, often pushing many to the brink of extinction [3]. The loss of sea fan corals caused by *Aspergillus sydowii* exemplifies the impact that fungal pathogens can have on marine ecosystems, with over 50% of the colony area being lost within a six-year span in Florida [4]. This phenomenon mirrors the broad impacts of emerging fungal diseases observed across terrestrial and freshwater habitats globally. For example, the white-nose syndrome, caused by the fungus *Pseudogymnoascus destructans,* has led to declines of over 75% in the populations of several endangered bat species in the United States [5]. Similarly, *Batrachochytrium dendrobatidis*, the pathogen responsible for chytridiomycosis, has caused widespread declines and, in some cases, the extinction of numerous amphibian species worldwide [6]. Meanwhile, the crayfish plague, caused by *Aphanomyces astaci,* is driving European and Asian freshwater crayfish species toward extinction [7,8].

Sea turtles are no exception to the impacts of emerging fungal diseases. While multiple factors have contributed to the decline of several populations globally [9–11], a newly described fungal disease, sea turtle egg fusariosis (hereafter referred to as STEF), has emerged as an important threat in recent years [12]. The disease is caused by two closely related species, *Fusarium falciforme* (hereafter *Ff*) and *Fusarium keratoplasticum* (hereafter *Fk*), which are part of the *Fusarium solani* species complex (FSSC) [12–14].

*Both Ff* and *Fk* have been linked to the failure of sea turtle and freshwater turtle eggs [14–16], as well as elevated clutch mortality rates [17]. This pathogenicity has been demonstrated under laboratory conditions by inoculating sea turtle eggs with pure *Fusarium* cultures, resulting in mortality rates of up to 83% among the tested eggs [17]. Both pathogens have been found to thrive in the nest environment, growing optimally at temperatures ranging from 27 to 32 ºC [14,18,19], which coincides with the optimal temperature range for embryo development [20]. Their ability to grow in such conditions poses a threat to embryo development, as severe infections can impede gas exchange, lower pH levels, deplete shell calcium, and ultimately lead to egg internal hyphal growth [17,21–23].

The presence of *Fusarium* species has been reported in the clutches of six out of the seven existing sea turtle species, except for the Kemp’s ridley (*Lepidochelys kempii*), which lacks sufficient information [24–33]. Additionally, *Fusarium* species have been found in the sand of loggerhead (*Caretta caretta*), green (*Chelonia mydas*), leatherback (*Dermochelys coriacea*), and hawksbill (*Eretmochelys imbricata*) nests at various locations around the world [26,28,34,35]. In Costa Rica, studies have reported potential occurrences of *Ff* and *Fk*. For instance, Sarmiento-Ramírez et al. [12] detected possible *Ff* and *Fk* isolates in leatherback failed eggs at Pacuare on the Caribbean coast. Similarly, on the Pacific coast, Bézy et al. [36] analyzed fungal diversity in olive ridley nest sand at Ostional National Wildlife Refuge and identified some sequences closely related to *Fk*.

Despite these studies, the identification of pathogenic *Fusarium* in sea turtle nests has primarily relied on single-locus analyses, with only a few exceptions. For example, Sarmiento-Ramírez et al. [12] used three loci in their studies across Ecuador, Colombia, Ascension Island (British Overseas Territory), Cape Verde, and Australia, while Hoh et al. [37] applied the same multi-locus approach in Malaysia. Since *Ff* and *Fk* are closely related within FSSC, single-locus analyses may be insufficiently informative or resolutive, potentially leading to inaccurate phylogenetic inferences [13,38,39]. Consequently, identifications based solely on a single locus should be regarded as potential or uncertain until more robust methods are employed.

In contrast, high-resolution phylogenetic techniques, such as multilocus sequence typing (MLST), which analyze three or more loci simultaneously, enhance the detection and discriminatory power for accurately identifying pathogenic *Fusarium* species. [13,40,41]. To this end, we conducted fungal isolations from failed sea turtle eggs and nest sand and used a MLST approach to ensure precise identification. Additionally, given the pathogenic potential of STEF-causing species and their threat to embryo development, it is essential to document their presence at important nesting sites. Therefore, we conducted this study across four key nesting beaches for green (*Chelonia* mydas) and leatherback (*Dermochelys coriacea*) turtles along the Pacific and Caribbean coasts of Costa Rica.

## Materials and methods

### Study areas

**Fig 1:**
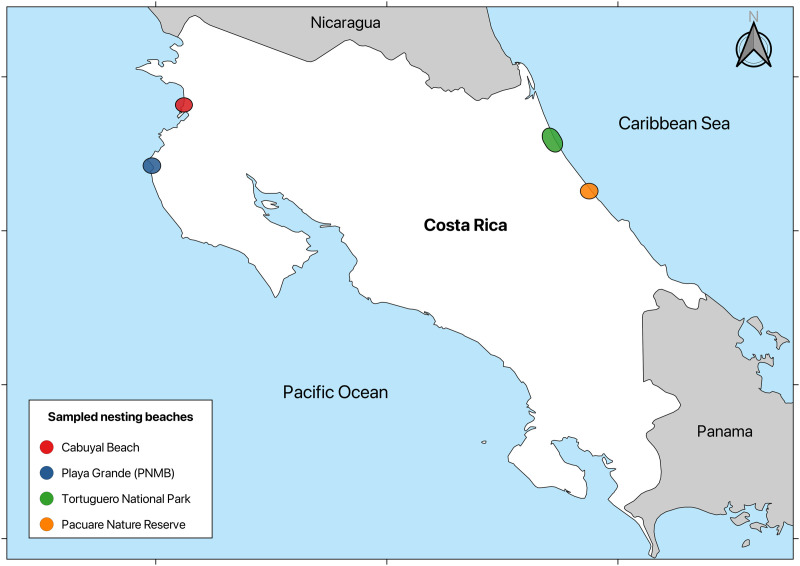
Sea turtle nesting beaches on the Pacific and Caribbean coasts assessed for the presence of pathogenic *Fusarium* species. Country boundaries were sourced from the Natural Earth dataset (1:10m scale, public domain) (https://www.naturalearthdata.com/downloads/10m-cultural-vectors/).

To assess the presence of *Fusarium* species that causes STEF, we selected four nesting beaches in Costa Rica that are recognized for their regional and international importance: (i) Playa Grande, within Las Baulas National Park, and (ii) Cabuyal are located on the Pacific coast, and (iii) Tortuguero National Park and (iv) Pacuare located on the Caribbean coast (Fig 1). Cabuyal [42] and Tortuguero National Park [43] beaches are key locations for green turtles, while Playa Grande [44] and Pacuare [45] beaches are important nesting places for leatherback turtles.

### Sample collection

Fieldwork was conducted between 2018 and 2023, aligned with the sea turtle nesting seasons on the Pacific (October to March for both green and leatherback turtles) [42,46] and Caribbean coasts (leatherback turtles: March to July; green turtles: June to November) [43,47]. The specific seasons sampled at each location are detailed in Table 1. During night patrols, we covered the entire beach length (only the northernmost 8 km for Tortuguero) and identified and marked nests during oviposition [48–51]. With the exception of Tortuguero, our sampling strategy involved collecting samples from every nest we successfully located. At Tortuguero, due to the high density of nests, we selected a spatially representative subset of nests. The final number of nests sampled per site was determined by annual nesting activity (i.e., the number of nests laid) and beach coverage (i.e., our ability to patrol the entire beach every night; Table 1).

**Table 1:** Number of nests sampled and nesting seasons during which sand and eggshell fragments were collected on the Pacific and Caribbean coasts.

Location^a^	Nests sampled	Sampled seasons^b^	
		Eggshell samples	Sand samples
Pacific Coast			
Cabuyal	13	2019/2020	2018/2020
Playa Grande (PNMB)	17	2018/2019	2018/2019
Caribbean Coast			
Tortuguero National Park	36	2019	2019
Pacuare	53 (10)	2018	2023

^a^For Pacuare, the number of sampled nests for sand is indicated in parentheses.

^b^Nesting season on the North Pacific extends from October to March, so sampled seasons are presented with two years separated by a slash (/).

**Fig 2:**
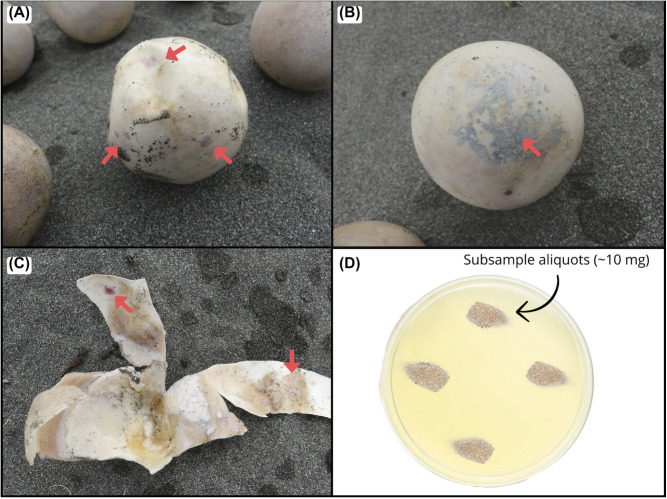
Eggs of *Chelonia mydas* (A) and *Dermochelys coriacea* (B), and internal view of one eggshell (C) with macroscopic signs of STEF (red arrows). Sand sample representation in a PGA Petri dish and placed at four different points to avoid colony overgrowth to isolate them (D).

We excavated nests two days after hatchling emergence. If emergence was missed or failed, nests were excavated after 65 days for green turtles [48,49] and after 70 days for leatherbacks (75 days at Pacuare due to longer incubation periods) [50,51]. During each excavation, we collected duplicate sand samples from approximately the middle of the clutch depth and stored them in labeled 1.5 ml Eppendorf tubes. We also collected 1 cm^2^ eggshell fragments from up to five failed eggs per clutch that exhibited characteristic signs of *Fusarium* infection, such as yellowish, reddish, and bluish spots (Fig. 2a-c). These fragments were rinsed with distilled water to remove organic residue, air-dried, and stored in labeled paper envelopes.

Following collection, we stored samples in a dry place at field temperature until the end of the fieldwork season. All samples were then shipped to the Molecular Systematics Laboratory of the Royal Botanical Garden (RJB-CSIC, Madrid, Spain). Upon arrival, samples were accessioned and stored at room temperature until processing. Fungal isolation procedures began within six months of the samples’ arrival in the lab. Subsequent DNA extraction, amplification, and sequencing were performed on isolates obtained from these cultures.

### Fungal isolation

#### Sand fungal isolation.

We isolated fungal colonies from the sand by dividing each sample into four sub-samples. Approximately 10 mg aliquots (Fig 2d) were spread onto Peptone Glucose Agar (PGA) plates (100 × 15 mm) supplemented with 100 μg/ml chloramphenicol to inhibit bacterial growth [35]. After incubating plates at 25°C for 2–5 days, we transferred hyphal fragments from colony margins to fresh PGA plates for isolation. These subcultures were incubated again at 25°C for 2–5 days. We obtained pure axenic cultures from the resulting colonies using the ring technique described by Cerenius et al. [52].

#### Eggshell fungal isolation.

We conducted fungal isolation from eggshells following Sarmiento-Ramírez et al. [12] by placing selected fragments on PGA plates containing ampicillin (100 mg/l) and incubating at 25°C for 2–5 days. We subculture the resulting fungal colonies using the ring technique to produce axenic cultures. All isolated strains from both sand and eggshell samples were deposited in the Royal Botanical Garden (RJB-CSIC, Madrid, Spain) culture collection.

### DNA extraction, PCR amplification, and sequencing

For DNA extraction, we cultured pure mycelium in liquid drops of peptone-glucose media (PG-l) for 1–2 days [17,53]. Mycelial fragments from drop culture samples were mechanically ruptured using a TissueLyser (Qiagen Iberia®, Madrid, Spain). We extracted genomic DNA using the E.Z.N.A® SP Plant DNA kit (Omega Biotek, Norcross, GA).

To implement the MLST approach, we used the following three locus primer pairs: (1) ITS5/ITS4 [54], which correspond to the internal transcribed spacer region of the nuclear ribosomal DNA (hereafter referred to as nrITS); (2) LR0R/LR6 [54,55], which corresponds to a portion of the large subunit ribosomal DNA that contains the domains D1 and D2 (hereafter referred to as LSU); (3) EF-1 and EF-2 [56], which correspond to a portion of the translation elongation factor 1-α (hereafter referred to as TEF1) (Table 2).

**Table 2:** Primers and DNA regions used in the phylogenetic analysis.

DNA region	Primer name	Primer sequence (5` → 3′)	Reference
ITS	ITS5 ITS4	GGAAGTAAAAGTCGTAACAAGG TCCTCCGCTTATTGATATGC	[54]
LSU	LR0R LR5	ACCCGCTGAACTTAAGC ATCCTGAGGGAAACTTC	[54,55]
TEF1	EF-1 EF-2	ATGGGTAAGGA(A/G)GACAAGAC GGA(G/A)GTACCAGT(G/C)ATCATGTT	[56]

We performed single-round PCR for each locus following Sarmiento-Ramírez et al. [14]. Amplification products were visualized on 1% agarose gel stained with SYBR® Safe DNA (Invitrogen®, Madrid, Spain), and positive amplicons were sequenced using an automated sequencer (MACROGEN, Inc. Madrid, Spain).

### Phylogenetic analyses

To initially identify the fungal isolates, the nrITS, LSU, and TEF1 sequences obtained from each isolate were compared with the sequences present in the GenBank database using the BLAST tool for rapid sequence similarity comparisons [57]. Sequences that belonged to the FSSC were further assembled, edited, and trimmed to exclude low-quality and primer-binding sites using Geneious Prime [58]. To determine the phylogenetic relationships of the obtained isolates, we used two phylogenetic approximations: Bayesian Inference (BI) and Maximum Likelihood (ML). Both analyses were initially conducted separately for each DNA region.

We first ran a preliminary ITS analysis to determine whether the obtained *Fusarium* isolates belonged to STEF-causing species. Prior to running this preliminary analysis, all sequences were automatically aligned with 149 GenBank sequences from the FSSC (derived from diverse hosts and environments), along with the *Fusarium incarnatum-equiseti* (FIESC) type sequence (NRRL26419), and a *Fusarium oxysporum* species complex (FOSC) reference sequence (199FUS) (S1 Table). Based on the preliminary results, we selected a subset of isolates that grouped within the STEF-causing species and performed their MLST analysis simultaneously with the same 149 FSSC GenBank sequences. In all analyses, we selected the *Geejayessia atrofusca* sequence (NRRL22316) as the outgroup according to previous studies [40,59].

We conducted BI analyses in MrBayes version 3.2.7 [60] using the high-performance computing platform of the CIPRES Science Gateway for phylogenetics [61]. To determine the best nucleotide substitution model for the DNA regions, we used JModelTest version 2.1.10 [62] under the Bayesian Information Criterion (BIC). We ran MrBayes using the Markov chain Monte Carlo (MCMC) method as described in Sarmiento-Ramírez et al. [12].

We conducted ML analyses using raxml-GUI version 2.0.10 [63]. For each dataset, the optimal nucleotide substitution model was determined using ModelTest-ng version 0.1.7 [64]. Nodal bootstrap support (BS) was assessed through 1000 non-parametric bootstrap replicates. We visualized and annotated the resulting single-gene phylogenetic trees using FigTree [65]. Then, we adjusted the root position and added the scale bar representing the average number of nucleotide substitutions per site.

Given congruent topologies from single-gene analyses, we concatenated the nrITS, LSU, and TEF1 for a more informative and resolutive analysis. We conducted combined analyses using MrBayes for the BI and raxml-GUI for ML, with the same parameters previously used in our single-locus approach. Final phylogenetic trees were visualized in FigTree and edited with Adobe Illustrator [66].

### Ethics statement

This research was approved by the Animal Research Ethics Committee of Comisión Nacional para la Gestión de la Biodiversidad (CONAGEBIO) and conducted under CONAGEBIO research permits (R-012–2019-OT-CONAGEBIO). Tempisque, Guanacaste, Tortuguero, and La Amistad Caribe Conservation Areas also granted research permits for the sea turtle projects. No anesthesia, euthanasia, or any kind of animal sacrifice is part of the study.

## Results

### Fungal isolation and rapid sequence similarity comparisons

We analyzed 408 eggshell fragments and 161 sand samples to determine the presence of STEF pathogenic species, obtaining 76 and 71 axenic cultures, respectively. Of these, we obtained 74 from Pacuare, 25 from Cabuyal Beach, 26 from Playa Grande, and 22 from Tortuguero National Park (Table 3). No fungal colonies grew from Tortuguero National Park eggshell fragments. BLAST analyses (>99% similarity values) indicated that 37% (n = 55) of isolates belonged to FSSC, while 4% (n = 6) corresponded to other species of the genus *Fusarium* other than the FSSC, such as *F. equiseti* and *F. oxysporum*. The remaining 59% (n = 86) belonged to other genera, including *Aspergillus, Acrophialophora*, *Chaetomium*, *Curvularia*, *Endocarpon*, *Hypocrea*, *Phytophytium*, *Trichoderma*, among others (S3 Table).

**Table 3:** Number of samples, isolates, *Fusarium falciforme* isolates (*Ff*), and *Fusarium keratoplasticum* isolates (*Fk*) from eggshells and sand of green and leatherback turtle nests.

Location	Sea turtle species	Sample type							
		Eggshell				Sand			
		Samples	Isolates	*Ff*	*Fk*	Samples	Isolates	*Ff*	*Fk*
Cabuyal	Green	105	5	1	0	30	20	10	0
Playa Grande (PNMB)	Leatherback	36	13	3	0	17	13	1	0
Tortuguero National Park	Green	100	0	0	0	60	22	6	0
Pacuare	Leatherback	67	58	10	15	20	16	1	0

### Preliminary ITS phylogenetic analyses

Our preliminary ITS analysis showed that of the isolates belonging to the FSSC, six grouped with *F. neocosmosporiellum*, two with *F. solani,* and 47 with the STEF-causing species *Ff* (n = 32) and *Fk* (n = 15) (S1 Fig). *Ff* was present in both eggshells and sand of sea turtle nests in Cabuyal Beach, Playa Grande, and Pacuare, as well as in the sand of Tortuguero National Park nests (Table). On the other hand, *Fk* was exclusively found in failed eggs from Pacuare (n = 15). Of the 47 STEF-causing isolates found, we selected 22 from both sand and eggshell samples to fully confirm their identity and presence in the studied locations with the MLST (GenBank accession numbers in S2 Table).

### MLST phylogenetic analyses

The best substitution models for the DNA regions were TIM2ef+I + G for ITS, K80 + I + G for LSU, and TIM2 + I + G for TEF1. While independent phylogenetic analyses of these DNA regions were consistent, they did not fully clarify all relationships within the FSSC. Topologies of all single-locus trees exhibited limited resolution, with multiple polytomies (e.g., clade 3; S2-S4 Figs), and low nodal support (PP < 0.95, BS < 70%). These nodal values fall below accepted thresholds [67,68] and are generally considered as weak support for the existence of a node or true clades.

**Fig 3:**
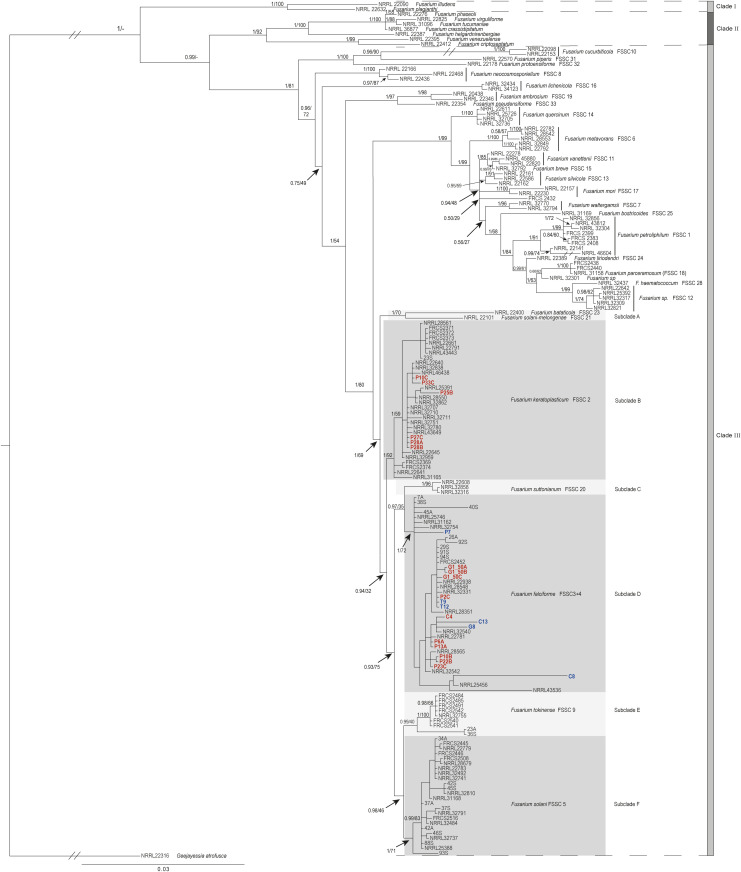
Consensus phylogenetic tree of *Fusarium Solani* Species Complex obtained by Bayesian inference. The dataset comprises the concatenation of the ITS, LSU, and TEF1 nDNA sequences, based on 1679 nucleotide positions from 149 GenBank sequences, 22 STEF-causing isolates selected for the analysis from failed eggs (bold red) and sand (bold blue), and the outgroup *Geejayessia atrofusca* (NRRL22316). Posterior probabilities (PP) and bootstrap support (BS) derived from the Bayesian Inference and Maximum Likelihood analyses, respectively, are represented on the branches (PP/BS). An interrupted branch (//) indicates its length has been reduced for representation purposes. The scale bar represents the average number of nucleotide substitutions per site. From the 22 STEF-causing isolates selected, those from Playa Grande start with G, Cabuyal with C, Pacuare with P, and Tortuguero with T.

The BI and ML phylogeny for the combined locus dataset had the same topology, branch lengths, and level of support for the given clades. For this reason, only the Bayesian tree showing PP and BS values is provided in our results (Fig 3). FSSC species grouped into three clades strongly supported by the BI and ML: clades I (PP = 1, BS = 100%), II (PP = 1, BS = 92%), and III (PP = 1, BS = 81%). Clade III comprised six key subclades (A-F), which corresponded to different FSSC species described in previous phylogenetic studies [13,40,59]. Subclade A included the *F. bataticola* (FSSC23) and *F. solani-melongenae* (FSSC21) sequences; Subclade B comprises *F. keratoplasticum* (FSSC 2) sequences; Subclade C included *F. suttonianum* (FSSC 20) sequences; Subclade D contained *F. falciforme* (FSSC 3 + 4) sequences; Subclade E included *Fusarium tokinense* (FSSC 9) sequences; and Subclade F comprises the *F. solani* (FSSC 5) sequences. Both BI and ML highly supported all subclades with the exception of subclade E, which received high support by BI (PP = 0.99), but low support by ML (BS = 40%). The MLST analysis fully confirmed the presence of the STEF-causing species. *Fk* was only found in eggshell samples of *D. coriacea* from Pacuare. All the other isolates from eggshells and sand were clustered within the *Ff* subclade in all locations (Fig 3).

## Discussion

This study provides the first documented presence of two pathogens linked to the emerging disease STEF in Costa Rica using a comprehensive three-locus genetic analysis. We detected the presence of *Ff* in the failed eggs and nesting sand of Cabuyal, Playa Grande, and Pacuare beaches, as well as in the nesting sand of Tortuguero. Additionally, we confirmed the presence of *Fk* in failed leatherback turtle eggs in Pacuare, corroborating earlier reports by Sarmiento-Ramírez et al. [14]. Furthermore, we successfully applied a new method for isolating species of *Fusarium* directly from the sand, yielding a total of 18 *Ff* isolates. Common techniques have relied on suspending the sand in water or saline solutions, followed by several serial dilutions before culturing fungal colonies [26,28,32,35,37,69], which makes the process lengthy and meticulous. With our approach, aliquots from the sand samples are placed directly on PGA with chloramphenicol. This method enables an immediate start to fungal incubation, thereby simplifying the procedure in terms of both time and effort while still yielding satisfactory results. Thus, this methodology may offer an alternative for isolating these pathogens from the sand in future studies.

Species within the FSSC are often generalists and opportunists [13,19]. *Ff* and *Fk* have been found to be associated with the biofilm of drainage holes, shower cabins, and sewage pipes [70–72]. Both species can act as plant and animal pathogens [70,72,73], however, they can also survive outside a host, and their spores can persist in the soil for several years [14,74]. On sea turtle nesting beaches, *Ff* and *Fk* have been detected across multiple regions worldwide [18]. In our study, we identified *Ff* in eggshells and sand at all study locations, except for Tortuguero. At this site, we were unable to isolate fungal colonies from the eggshells, likely because samples were not thoroughly cleaned and retained organic matter that promoted decomposition and bacterial overgrowth. However, this absence in eggshells from Tortuguero does not exclude the presence of the pathogen in the environment. *Ff* can persist in the sand as a saprotroph [14]. Consequently, plant material and other organic particles on the beaches may serve as reservoirs for this pathogen, facilitating its widespread presence in all the studied areas.

On the other hand, we detected *Fk* exclusively on the Caribbean coast, in failed leatherback eggs at Pacuare. Interestingly, this species has already been reported on both coasts, the Caribbean and Pacific, in countries near Costa Rica. For example, Sarmiento-Ramírez et al. [14] found *Fk* in failed leatherback eggs from the Caribbean of Colombia and in failed green (*Chelonia mydas*) and hawksbill (*Eretmochelys imbricata*) eggs from the Pacific coast of Ecuador [14]. Additionally, some potential *Fk* isolates have also been found in olive ridley (*Lepidochelys olivacea*) failed eggs and nesting sand along the Pacific coast of Colombia [32]. Given this geographical proximity, it is likely that *Fk* occurs on the studied beaches and other locations across both coasts. On the other hand, climatic conditions and sand composition could influence *Fusarium* distribution. For instance, Pacuare and Tortuguero are characterized by black sand beaches with low calcium content [75], whereas the sand in Cabuyal and Playa Grande has higher carbonate levels [76]. Therefore, further studies are necessary to gain a deeper understanding of the factors that influence its occurrence.

We also isolated other *Fusarium* species, such as *F. oxysporum* in Pacuare and Tortuguero, and *F. solani* in Pacuare. These fungi have been linked to skin lesions in loggerhead turtles in the Mediterranean Sea [77,78], and are also associated with failed sea turtle eggs in various locations worldwide [17,27,79]. Thus, they could pose a similar threat to turtles and embryonic development in Costa Rica, and further studies could assess their pathogenicity. In addition, *F. neocosmosporiellum* was isolated from eggs and sand at Cabuyal and Playa Grande. This fungus is a known human pathogen that causes skin and pulmonary lesions [80]. It has only been previously reported in the cloacal microbial community of loggerhead turtles in the Adriatic Sea [81], and we report it, for the first time, in association with failed sea turtle eggs. However, its role as a pathogen for sea turtles remains unclear. Finally, we isolated *F. equiseti* from the eggshells of leatherback turtles in Pacuare. This saprophytic fungus has a cosmopolitan distribution and is generally associated with decaying plant material and as a crop pathogen [82]. Even though there are no reports associated to sea turtles, its presence in nests warrants further study, especially given that the FIESC is one of the most important clades of veterinary relevance due to its mycoses-associated species [83].

Besides FSSC and other species from the genus *Fusarium*, we isolated other fungal species that can act as plant, animal, or human pathogens (S3 Table). Among them, there were species of the genus *Aspergillus* in all studied nesting beaches (*A. flavus*, *A. hortae*, *A. quadilineatus*, *A. sidowii*, *A. tamarii*, *and A. terreus*) that were found in both eggshells and sand. These species have been commonly found on sea turtle eggs and are known to produce mycotoxins that could negatively influence embryo development [84]. These toxins, as well as those produced by the *Fusarium* pathogens, may interfere with gas exchange, deplete shell calcium, and damage the allantois and embryonic tissue, leading to embryo failure [35,84,85].

### Implications for conservation

It is important to highlight that the presence of *Fusarium* does not always result in the development of the disease. For example, pathogenic *Fusarium species* can be isolated from asymptomatic eggs [17] and be present in nests with high hatching success [69]. However, as is characteristic of emerging fungal diseases like STEF, disease incidence is often low until specific factors, such as climate, environmental changes, or host conditions, trigger virulence [1]. Carbon availability, for instance, could play a role in shaping the pathogenicity of *Fusariu*m. As shown under laboratory conditions, high carbon content can acidify the host environment and eggshell, while low carbon content may result in alkalinization [23]. These changes can modulate the activation of genes associated with *Fusarium*’s pathogenic mechanisms to colonize the eggs [86]. Furthermore, under specific conditions, such as increased clay or silt content in the sand composition, the pathogenicity of *Fusarium* can escalate, resulting in severe outcomes like 0% hatch success, as observed in numerous loggerhead turtle nests on Boa Vista Island, Cape Verde [17].

Evidence supporting the pathogenicity of *Fusarium* has been shown under controlled conditions. Sarmiento-Ramírez et al. [13] and Martínez-Ríos et al. [19] found that exposing eggs of loggerhead sea turtles (*C. caretta*) and red-eared slider turtles (*Trachemys scripta*), respectively, to pure *Fusarium* cultures resulted in a mortality rate of >80%, significantly higher than controls. This shows a pathogenic, not saprophytic, relationship between the fungus and the turtle eggs. Under global change scenarios, the incidence and severity of STEF could increase, posing an additional threat to sea turtle populations. The consequences of STEF could be even greater for specific populations, such as the leatherback turtles in the Eastern Tropical Pacific region, where their conservation status is critically endangered, and their populations are declining [87].

This study represents the first survey to identify fungal pathogens across the studied beaches, confirming the widespread distribution of *Ff* and *Fk* using a multilocus approach. Given the capacity of *Fusarium* species to persist in sand [14,74] and potential pathogenicity, future conservation efforts could integrate quantitative *Fusarium* load studies (e.g., through qPCR, culture-based counts) with nest environmental variables (e.g., sand composition, temperature, and humidity) to determine their effect on nest success. This would help determine whether higher fungal concentrations correlate with reduced hatching success and under what conditions. Additionally, we identified other fungal species that could potentially become pathogenic (S3 Table). Quantifying their virulence through controlled infection assays, as has been done with *Fusarium*, is critical to understanding their potential to become pathogenic.

## Supporting information

S1 Fig Preliminary Bayesian ITS tree of all FSSC and other species from the genus *Fusarium* isolated in the study. The tree is based on 511 nucleotides from 149 GenBank sequences, all *Fusarium sp.* found in this study in failed eggs (bold red) and sand (bold blue), and the outgroup *Geejayessia atrofusca* (NRRL22316). *F. oxysporum* (GenBank type sequence NRRL24429) and *F. incarnatum-equiseti* (GenBank sequence 199FUS) Species Complexes are also included. Posterior probabilities (PP) and bootstrap support (BS) derived from the Bayesian Inference and Maximum Likelihood analyses respectively, are represented on the branches (PP/BS). An interrupted branch (//) indicates its length has been reduced for representation purposes. The scale bar represents the average number of nucleotide substitutions per site. From the *Fusarium sp.* found in this study, the Playa Grande code starts with G, Cabuyal with C, Pacuare with P, and Tortuguero with T.
(TIF)

S2 Fig Bayesian ITS tree of *Fusarium Solani* Species Complex. The tree is based on 504 nucleotides from 149 GenBank sequences, 22 STEF-causing isolates selected for the analysis from failed eggs (bold red) and sand (bold blue), and the outgroup *Geejayessia atrofusca* (NRRL22316). Posterior probabilities (PP) and bootstrap support (BS) derived from the Bayesian Inference and Maximum Likelihood analyses respectively, are represented on the branches (PP/BS). An interrupted branch (//) indicates its length has been reduced for representation purposes. The scale bar represents the average number of nucleotide substitutions per site. From the 22 STEF-causing isolates selected in the analysis, the Playa Grande code starts with G, Cabuyal with C, Pacuare with P, and Tortuguero with T.
(TIF)

S3 Fig Bayesian LSU tree of Fusarium Solani Species Complex. The tree is based on 473 nucleotides from 149 GenBank sequences, 22 STEF-causing isolates selected for the analysis from failed eggs (bold red) and sand (bold blue), and the outgroup *Geejayessia atrofusca* (NRRL22316). Posterior probabilities (PP) and bootstrap support (BS) derived from the Bayesian Inference and Maximum Likelihood analyses respectively, are represented on the branches (PP/BS). An interrupted branch (//) indicates its length has been reduced for representation purposes. The scale bar represents the average number of nucleotide substitutions per site. From the 22 STEF-causing isolates selected, the Playa Grande code starts with G, Cabuyal with C, Pacuare with P, and Tortuguero with T.
(TIF)

S4 Fig Bayesian TEF1 tree of Fusarium Solani Species Complex. The tree is based on 702 nucleotides from 149 GenBank sequences, 22 STEF-causing isolates selected for the analysis from failed eggs (bold red) and sand (bold blue), and the outgroup *Geejayessia atrofusca* (NRRL22316). Posterior probabilities (PP) and bootstrap support (BS) derived from the Bayesian Inference and Maximum Likelihood analyses respectively, are represented on the branches (PP/BS). An interrupted branch (//) indicates its length has been reduced for representation purposes. The scale bar represents the average number of nucleotide substitutions per site. From the 22 STEF-causing isolates selected in this study (bold), Playa Grande code starts with G, Cabuyal with C, Pacuare with P, and Tortuguero with T.
(TIF)

S1 Table GenBank sequences of ITS, LSU, and EFa1 DNA regions from the *Fusarium solani species complex* included in the phylogenetic analysis. (DOCX)

S2 Table Selected STEF-causing isolates collected from eggshells and nest sand for the phylogenetic analysis. (DOCX)

S3 Table FSSC members and other fungal species isolates obtained from *C. mydas* and *D. coriacea* eggshells and nest sand in all study areas. (DOCX)
