# Supplementary figures and images for: Isolation and molecular identification of pathogens causing sea turtle egg fusariosis in key nesting beaches in Costa Rica
Source: PLoS One. 2025 Sep 25;20(9):e0333280. doi: 10.1371/journal.pone.0333280 (PMC12463207; doi:10.1371/journal.pone.0333280)

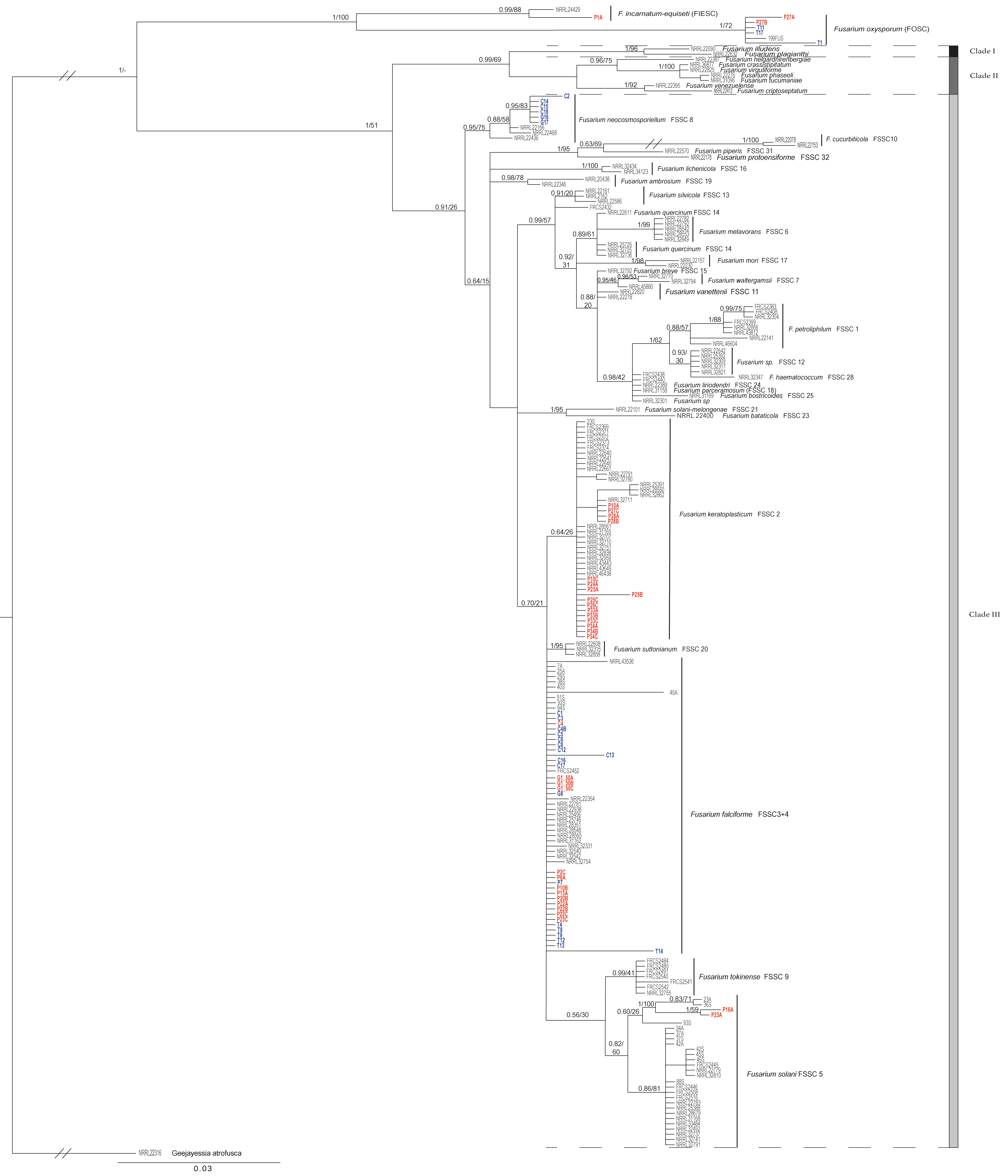

Supplement: S1 Fig — The tree is based on 511 nucleotides from 149 GenBank sequences, all Fusarium sp. found in this study in failed eggs (bold red) and sand (bold blue), and the outgroup Geejayessia atrofusca (NRRL22316). F. oxysporum (GenBank type sequence NRRL24429) and F. incarnatum-equiseti (GenBank sequence 199FUS) Species Complexes are also included. Posterior probabilities (PP) and bootstrap support (BS) derived from the Bayesian Inference and Maximum Likelihood analyses respectively, are represented on the branches (PP/BS). An interrupted branch (//) indicates its length has been reduced for representation purposes. The scale bar represents the average number of nucleotide substitutions per site. From the Fusarium sp. found in this study, the Playa Grande code starts with G, Cabuyal with C, Pacuare with P, and Tortuguero with T. (TIF) [file pone.0333280.s001.tif]

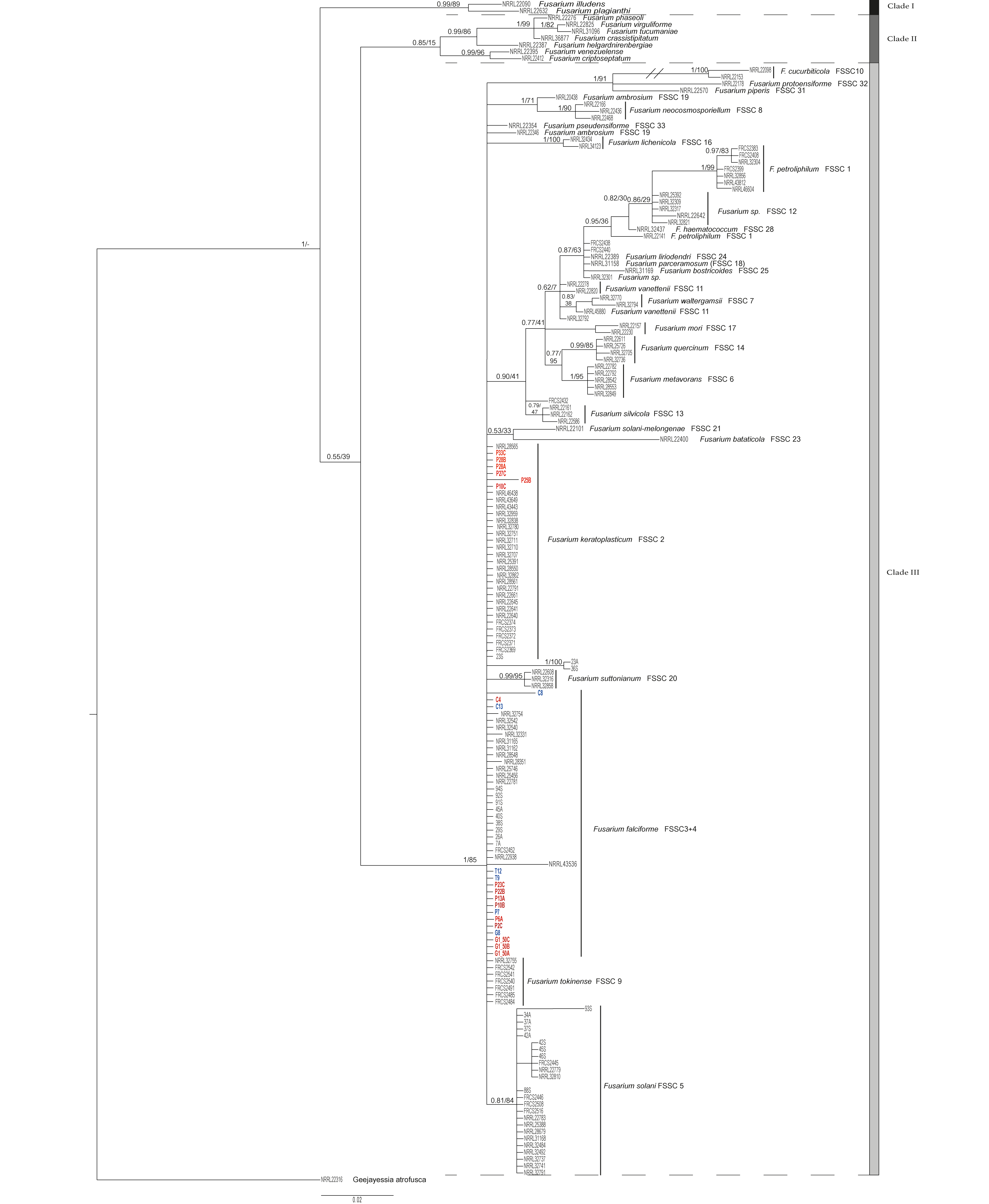

Supplement: S2 Fig — The tree is based on 504 nucleotides from 149 GenBank sequences, 22 STEF-causing isolates selected for the analysis from failed eggs (bold red) and sand (bold blue), and the outgroup Geejayessia atrofusca (NRRL22316). Posterior probabilities (PP) and bootstrap support (BS) derived from the Bayesian Inference and Maximum Likelihood analyses respectively, are represented on the branches (PP/BS). An interrupted branch (//) indicates its length has been reduced for representation purposes. The scale bar represents the average number of nucleotide substitutions per site. From the 22 STEF-causing isolates selected in the analysis, the Playa Grande code starts with G, Cabuyal with C, Pacuare with P, and Tortuguero with T. (TIF) [file pone.0333280.s002.tif]

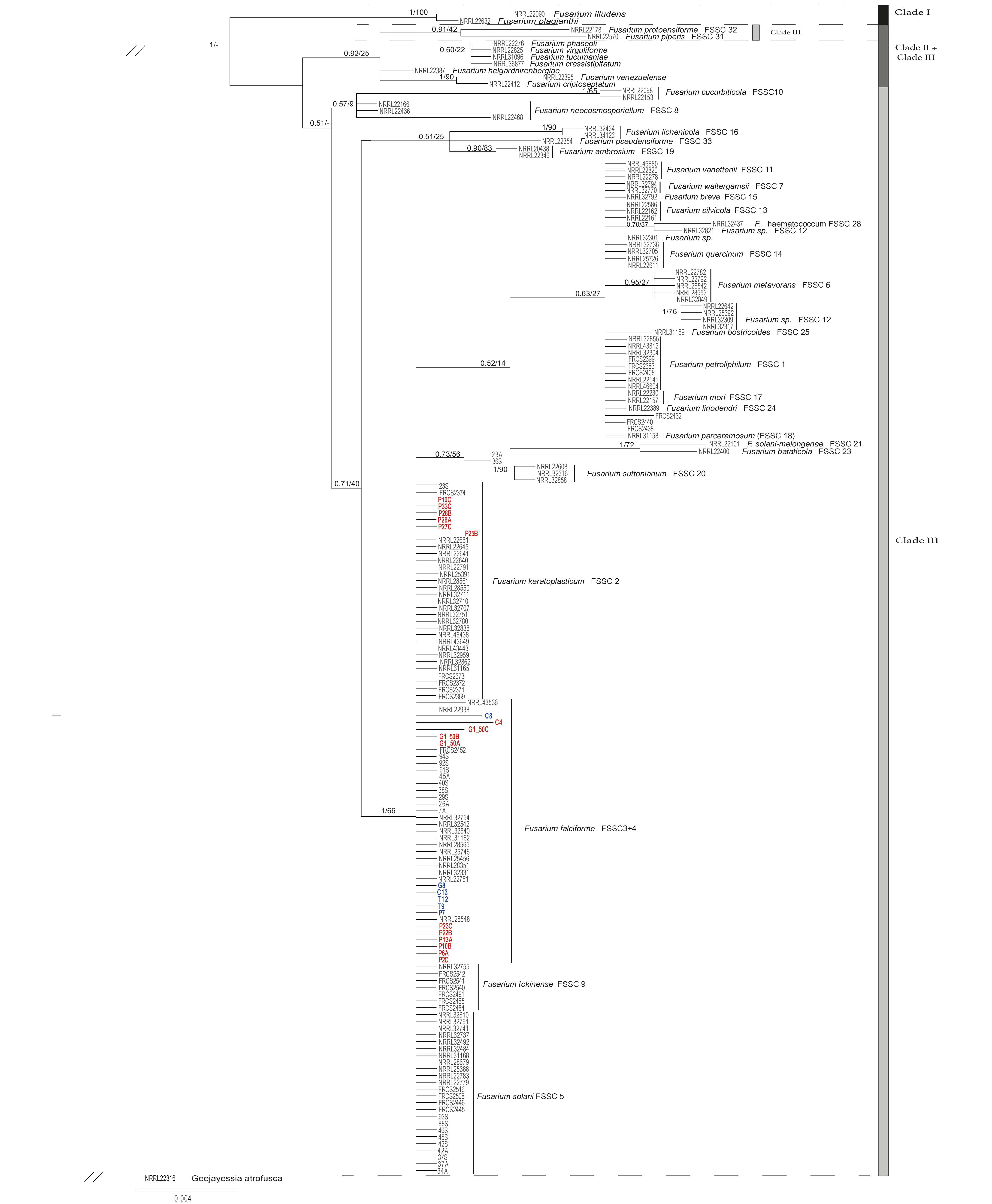

Supplement: S3 Fig — The tree is based on 473 nucleotides from 149 GenBank sequences, 22 STEF-causing isolates selected for the analysis from failed eggs (bold red) and sand (bold blue), and the outgroup Geejayessia atrofusca (NRRL22316). Posterior probabilities (PP) and bootstrap support (BS) derived from the Bayesian Inference and Maximum Likelihood analyses respectively, are represented on the branches (PP/BS). An interrupted branch (//) indicates its length has been reduced for representation purposes. The scale bar represents the average number of nucleotide substitutions per site. From the 22 STEF-causing isolates selected, the Playa Grande code starts with G, Cabuyal with C, Pacuare with P, and Tortuguero with T. (TIF) [file pone.0333280.s003.tif]

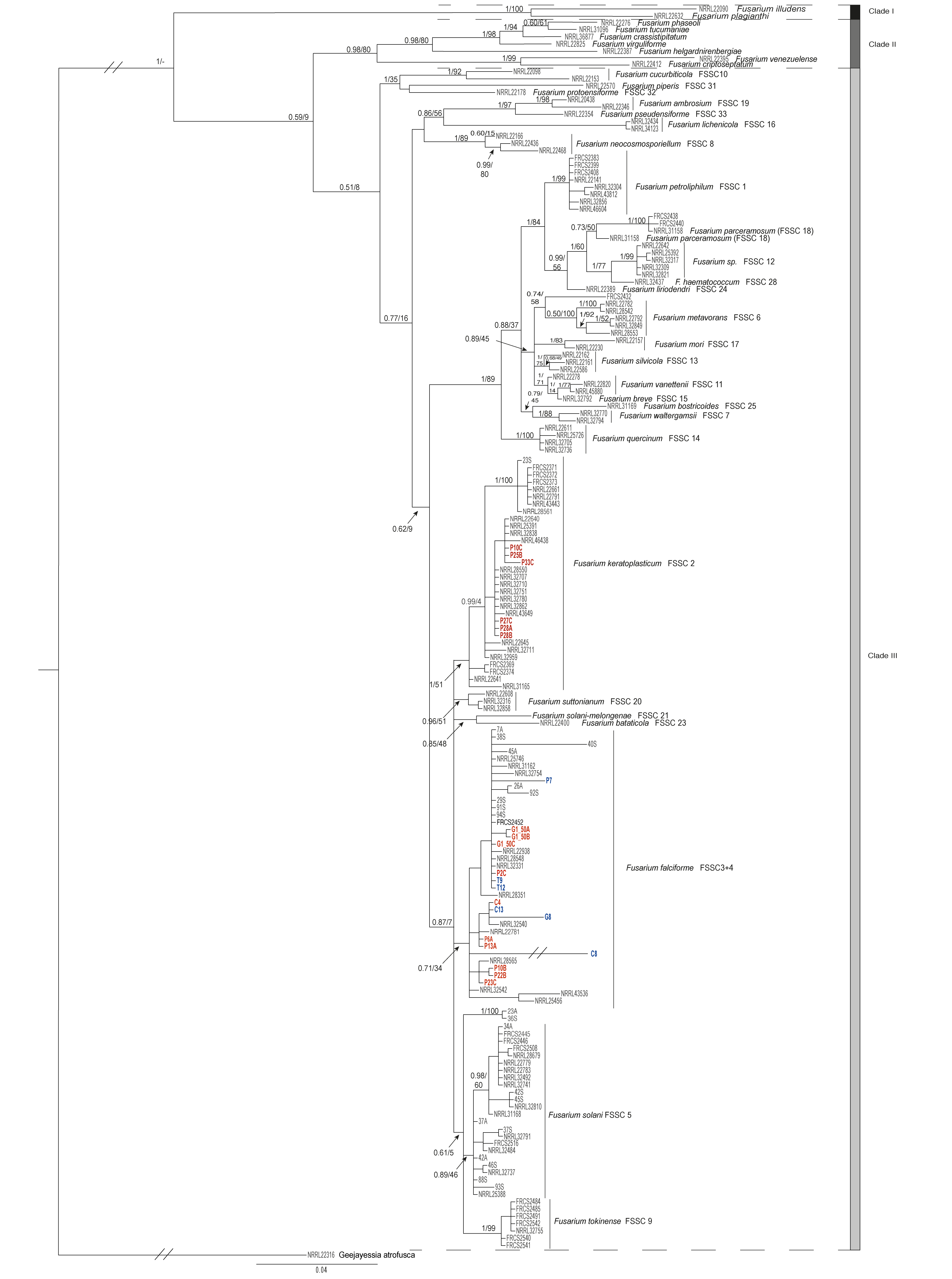

Supplement: S4 Fig — The tree is based on 702 nucleotides from 149 GenBank sequences, 22 STEF-causing isolates selected for the analysis from failed eggs (bold red) and sand (bold blue), and the outgroup Geejayessia atrofusca (NRRL22316). Posterior probabilities (PP) and bootstrap support (BS) derived from the Bayesian Inference and Maximum Likelihood analyses respectively, are represented on the branches (PP/BS). An interrupted branch (//) indicates its length has been reduced for representation purposes. The scale bar represents the average number of nucleotide substitutions per site. From the 22 STEF-causing isolates selected in this study (bold), Playa Grande code starts with G, Cabuyal with C, Pacuare with P, and Tortuguero with T. (TIF) [file pone.0333280.s004.tif]
