# Supplementary material for: Isolation and molecular identification of pathogens causing sea turtle egg fusariosis in key nesting beaches in Costa Rica
Source: PLoS One. 2025 Sep 25;20(9):e0333280. doi: 10.1371/journal.pone.0333280 (PMC12463207; doi:10.1371/journal.pone.0333280)
Supplement: S1 Table — (DOCX) [file pone.0333280.s005.docx]

**S1 Table.** GenBank sequences of ITS, LSU, and EFa1 DNA regions from the *Fusarium solani* species complex included in the phylogenetic analysis.

| **Clade** | **Subclade** | **Species (anamorph)** | **Isolate** | **GenBank Accession** | | | **Host (family: species)** | **Origin** |
| --- | --- | --- | --- | --- | --- | --- | --- | --- |
|  |  |  |  | ITS nrDNA | LSU nrDNA | TEF1 nDNA |  |  |
| Outgroup |  | *Geejayessia atrofusca* | NRRL22316 | AF178423 | AF178392 | AF178361 | Staphyleaceae: *Staphylea trifolia* | USA |
| FIESC* |  | *F. incarnatum-equiseti* | NRRL26419 | NR_121457 |  |  |  |  |
| FOSC* |  | *Fusarium oxysporum* | 199FUS | MW390951 |  |  |  |  |
| I |  | *F. illudens* | NRRL22090 | AF178417 | AF178386 | AF178354 | Lauraceae: *Beilschmiedia tawa* | New Zealand |
| I |  | *F. plagianthi* | NRRL22632 | AF178417 | AF178386 | AF178354 | Malvaceae: *Hoheria glabrata* | New Zealand |
| II |  | *F. phaseoli* | NRRL22276 | EF408520 | EU329668 | EF408415 | Fabaceae: *Phaseolus vulgaris* | USA |
| II |  | *F. tucumaniae* | NRRL31096 | EF408523 | GU170656 | GU170636 | Fabaceae: *Glycine max* | Argentina |
| II |  | *F. crassistipitatum* | NRRL36877 | FJ240376 | FJ240376 | FJ240351 | Fabaceae: *Glycine max* | Argentina |
| II |  | *F. virguliforme* | NRRL22825 | AF178419 | AF178388 | GU170635 | Fabaceae: *Glycine max* | USA |
| II |  | *F. helgardnirenbergiae* | NRRL22387 | AF178403 | AF178372 | AF178339 | Bark | French Guiana |
| II |  | *F. venezuelense* | NRRL22395 | AF178405 | AF178374 | AF178341 | Bark | Venezuela |
| II |  | *F. criptoseptatum* | NRRL22412 | AF178414 | AF178383 | AF178351 | Bark | French Guiana |
| III |  | *F. cucurbiticola* | NRRL22098 | DQ094301 | DQ236243 | AF178327 | *Cucurbitacea: Cucurbita sp.* | USA |
| III |  | *F. cucurbiticola* | NRRL22153 | AF178410 | DQ236344 | AF178346 | *Cucurbitacea: Cucurbita sp.* | USA |
| III |  | *F. piperis* | NRRL22570 | AF178422 | AF178391 | AF178360 | Piperaceae: *Piper nigrum* | Brazil |
| III |  | *F. protoensiforme* | NRRL22178 | DQ094313 | DQ236355 | AF178334 | Dicot tree | Venezuela |
| III |  | *F. neocosmosporiellum* | NRRL22166 | DQ094319 | DQ23636 | AF178350 | Heteroderidae: *Heterodera glycines* | USA |
| III |  | *F. neocosmosporiellum* | NRRL22468 | DQ094318 | DQ236260 | AF178349 | Fabaceae: *Arachis hypogaea* | Guiana |
| III |  | *F. neocosmosporiellum* | NRRL22436 | DQ094317 | DQ236259 | AY381144 | Hominidae: *Homo sapiens* | South Africa |
| III |  | *F. lichenicola* | NRRL32434 | DQ094444 | DQ236486 | DQ246977 | Hominidae: *Homo sapiens* | Germany |
| III |  | *F. lichenicola* | NRRL34123 | DQ094645 | DQ236687 | DQ247192 | Hominidae: *Homo sapiens* | India |
| III |  | *F. psudensiforme* | NRRl22354 | DQ094316 | DQ236358 | AF178338 | Bark | French Guiana |
| III |  | *F. ambrosium* | NRRL20438 | DQ094315 | AF178366 | AF178332 | Theaceae: *Camellia sinensis* | India |
| III |  | *F. ambrosium* | NRRL22346 | EU329669 | EU329669 | FJ240350 | Theaceae: *Camellia sinensis* | India |
| III |  | *F. quercinum* | NRRL22611 | DQ094326 | DQ236368 | DQ246841 | Hominidae: *Homo sapiens* | USA |
| III |  | *F. quercinum* | NRRL25726 | DQ094345 | DQ236387 | DQ246863 | Hominidae: *Homo sapiens* | Germany |
| III |  | *F. quercinum* | NRRL32705 | DQ094488 | DQ236530 | DQ247025 | Hominidae: *Homo sapiens* | USA |
| III |  | *F. quercinum* | NRRL32736 | DQ094517 | DQ236559 | DQ247056 | Hominidae: *Homo sapiens* | USA |
| III |  | *F. metavorans* | NRRL22782 | EU329670 | EU329670 | DQ246850 | Hominidae: *Homo sapiens* | Spain |
| III |  | *F. metavorans* | NRRL28542 | EU329675 | EU329675 | DQ246883 | Hominidae: *Homo sapiens* | USA |
| III |  | *F. metavorans* | NRRL28553 | EU329676 | EU329676 | DQ246894 | Hominidae: *Homo sapiens* | USA |
| III |  | *F. metavorans* | NRRL32849 | EU329682 | EU329682 | DQ247155 | Hominidae: *Homo sapiens* | USA |
| III |  | *F. metavorans* | NRRL22792 | EU329671 | EU329671 | DQ246854 | Hominidae: *Homo sapiens* | USA |
| III |  | *F. vanettenii* | NRRL22278 | DQ094309 | DQ236251 | AF178337 | Fabaceae: *Pisum sativum* | USA |
| III |  | *F. vanettenii* | NRRL45880 | EU329689 | EU329689 | FJ240352 | Fabaceae: *Pisum sativum* | USA |
| III |  | *F. vanettenii* | NRRL22820 | DQ094310 | DQ236352 | AF178355 | Fabaceae: *Glycine max* | USA |
| III |  | *F. breve* | NRRL32792 | DQ094561 | DQ236603 | DQ247101 | Hominidae: *Homo sapiens* | Japan |
| III |  | *F. mori* | NRRL22157 | DQ094306 | DQ094306 | AF178359 | *Moraceae: Morus alba* | Japan |
| III |  | *F. mori* | NRRL22230 | DQ094305 | DQ236347 | AF178358 | *Moraceae: Morus alba* | Japan |
| III |  | *F. silvivola* | NRRL22161 | DQ094311 | DQ236353 | AF178330 | Fabaceae: Robinea pseudoacacia | Japan |
| III |  | *F. silvivola* | NRRL22162 | EU329667 | EU329667 | DQ247561 | Fabaceae: Robinea pseudoacacia | Japan |
| III |  | *F. silvivola* | NRRL22586 | DQ094312 | DQ236354 | AF178353 | Fabaceae: Robinea pseudoacacia | USA |
| III |  | *Fusarium sp.* | FRCS2432 | JN235326 | JN235326 | JN235756 | Plumbing drains | USA |
| III |  | *F. waltergamsii* | NRRL32770 | DQ094544 | DQ236586 | DQ247083 | Hominidae: *Homo sapiens* | USA |
| III |  | *F. waltergamsii* | NRRL32794 | DQ094563 | DQ236605 | DQ247103 | *Humidifier coolant* | USA |
| III |  | *F. bostricoides* | NRRL31169 | DQ094396 | DQ236438 | DQ246923 | Hominidae: *Homo sapiens* | USA |
| III |  | *F. petroliphilum* | NRRL32856 | EU329683 | EU329683 | DQ247161 | Plaster from ceiling | USA |
| III |  | *F. petroliphilum* | NRRL43812 | EF453205 | EF453205 | EF453054 | Contact lens solution | USA |
| III |  | *F. petroliphilum* | NRRL32304 | DQ094402 | DQ236444 | DQ246932 | Hominidae: *Homo sapiens* | USA |
| III |  | *F. petroliphilum* | FRCS2399 | JN235318 | JN235318 | JN235748 | Plumbing drains | USA |
| III |  | *F. petroliphilum* | FRCS2383 | JN235243 | JN235243 | JN235673 | Plumbing drains | USA |
| III |  | *F. petroliphilum* | FRCS2408 | JN235260 | JN235260 | JN235690 | Plumbing drains | USA |
| III |  | *F. petroliphilum* | NRRL22141 | DQ094307 | DQ236249 | AF178329 | Cucurbitaceae: *Cucurbita sp.* | New Zealand |
| III |  | *F. petroliphilum* | NRRL46604 | GU170649 | GU170649 | GU170629 | Hominidae: *Homo sapiens* | Italy |
| III |  | *F. liriodendri* | NRRL22389 | DQ094314 | DQ236356 | AF178340 | *Magnoliaceae: Liriodendron tulipfera* | USA |
| III |  | *Fusarium sp.* | FRCS2438 | JN235324 | JN235324 | JN235754 | Plumbing drains | USA |
| III |  | *Fusarium sp.* | FRCS2440 | JN235325 | JN235325 | JN235755 | Plumbing drains | USA |
| III |  | *F. parceramosum* | NRRL31158 | DQ094389 | DQ236431 | DQ246916 | Hominidae: *Homo sapiens* | USA |
| III |  | *Fusarium sp.* | NRRL32301 | EU329677 | EU329677 | DQ246928 | Hominidae: *Homo sapiens* | USA |
| III |  | *F. haematococcum* | NRRL32437 | DQ094446 | DQ236488 | DQ246979 | Hominidae: *Homo sapiens* | Switzerland |
| III |  | *Fusarium sp.* | NRRL22642 | DQ094329 | DQ236371 | DQ246844 | Prawn | Japan |
| III |  | *Fusarium sp.* | NRRL25392 | EU329672 | EU329672 | DQ246861 | Nephropidae: Loabster | USA |
| III |  | *Fusarium sp.* | NRRL32317 | DQ094414 | DQ236456 | DQ246945 | Tree fish eye | USA |
| III |  | *Fusarium sp.* | NRRL32309 | DQ094407 | DQ236449 | DQ246937 | Hominidae: *Homo sapiens* | USA |
| III |  | *Fusarium sp.* | NRRL32821 | DQ094587 | DQ236629 | DQ247128 | *Turtle egg* | USA |
| III | A | *F. bataticola* | NRRL22400 | DQ094303 | DQ236345 | AF178343 | Convulvulacea: *Ipomoea batatas* | USA |
| III | B | *F. solani-melongenae* | NRRL22101 | AF178398 | AF178367 | AF178333 | Cotton duck cloth | Panama |
| III | C | *F. keratoplasticum* | 23S | MT266933 | MT267276 | MT307320 | Emydidae: *Trachemys scripta* | Spain |
| III | C | *F. keratoplasticum* | FRCS2371 | JN235215 | JN235215 | JN235645 | Plumbing drains | USA |
| III | C | *F. keratoplasticum* | FRCS2372 | JN235220 | JN235220 | JN235650 | Plumbing drains | USA |
| III | C | *F. keratoplasticum* | FRCS2373 | JN235143 | JN235143 | JN235573 | Plumbing drains | USA |
| III | C | *F. keratoplasticum* | NRRL22661 | NRRL22661 | NRRL22661 | NRRL22661 | Hominidae: *Homo sapiens* | Japan |
| III | C | *F. keratoplasticum* | NRRL22791 | DQ094337 | DQ236379 | DQ246853 | Iguana | Unites Kingdom |
| III | C | *F. keratoplasticum* | NRRL43443 | EF453082 | EF453082 | EF452930 | Hominidae: *Homo sapiens* | Italy |
| III | C | *F. keratoplasticum* | NRRL28561 | DQ094375 | DQ236417 | DQ246902 | Hominidae: *Homo sapiens* | USA |
| III | C | *F. keratoplasticum* | NRRL22640 | DQ094327 | DQ236369 | DQ247627 | Hominidae: *Homo sapiens* | Argentina |
| III | C | *F. keratoplasticum* | NRRL32838 | EU329681 | EU329681 | DQ247144 | Hominidae: *Homo sapiens* | USA |
| III | C | *F. keratoplasticum* | NRRL46438 | GU170644 | GU170644 | GU170624 | Hominidae: *Homo sapiens* | Italy |
| III | C | *F. keratoplasticum* | NRRL25391 | DQ094343 | DQ236385 | DQ246860 | Crangonidae: Shrimp | USA |
| III | C | *F. keratoplasticum* | NRRL28550 | DQ094365 | DQ236407 | DQ246891 | Hominidae: *Homo sapiens* | USA |
| III | C | *F. keratoplasticum* | NRRL32862 | DQ094621 | DQ236663 | DQ247167 | Hominidae: *Homo sapiens* | USA |
| III | C | *F. keratoplasticum* | NRRL32707 | DQ094490 | DQ236532 | DQ247027 | Hominidae: *Homo sapiens* | USA |
| III | C | *F. keratoplasticum* | NRRL32710 | DQ094492 | DQ236534 | DQ247030 | Hominidae: *Homo sapiens* | USA |
| III | C | *F. keratoplasticum* | NRRL32751 | DQ094531 | DQ236573 | DQ247070 | Hominidae: *Homo sapiens* | USA |
| III | C | *F. keratoplasticum* | NRRL32780 | DQ094551 | DQ236593 | DQ247090 | Hominidae: *Homo sapiens* | USA |
| III | C | *F. keratoplasticum* | NRRL43649 | EU329687 | EU329687 | EF452980 | Hominidae: *Homo sapiens* | USA |
| III | C | *F. keratoplasticum* | NRRL22645 | DQ094330 | DQ236372 | DQ246845 | Crangonidae: Shrimp | USA |
| III | C | *F. keratoplasticum* | NRRL32711 | DQ094493 | DQ236535 | DQ247031 | Hominidae: *Homo sapiens* | USA |
| III | C | *F. keratoplasticum* | NRRL32959 | DQ094632 | DQ236674 | DQ247178 | Hominidae: *Homo sapiens* | USA |
| III | C | *F. keratoplasticum* | FRCS2369 | JN235152 | JN235152 | JN235582 | Plumbing drains | USA |
| III | C | *F. keratoplasticum* | FRCS2374 | JN235152 | JN235152 | JN235582 | Plumbing drains | USA |
| III | C | *F. keratoplasticum* | NRRL22641 | DQ094328 | DQ236370 | DQ246843 | Hominidae: *Homo sapiens* | Nigeria |
| III | C | *F. keratoplasticum* | NRRL31165 | DQ094394 | DQ236436 | DQ246921 | Hominidae: *Homo sapiens* | USA |
| III | D | *F. suttonianum* | NRRL22608 | DQ094323 | DQ236365 | DQ246838 | Hominidae: *Homo sapiens* | USA |
| III | D | *F. suttonianum* | NRRL32316 | DQ094413 | DQ236455 | DQ246944 | Hominidae: *Homo sapiens* | USA |
| III | D | *F. suttonianum* | NRRL32858 | DQ094617 | DQ236659 | DQ247163 | Hominidae: *Homo sapiens* | USA |
| III | E | *F. falciforme* | 7A | MT266932 | MT267290 | MT307308 | Emydidae: *Trachemys scripta* | Spain |
| III | E | *F. falciforme* | 26A | MT266935 | MT267281 | MT307306 | Emydidae: *Trachemys scripta* | Spain |
| III | E | *F. falciforme* | 92S | MT266937 | MT267277 | MT307309 | Emydidae: *Trachemys scripta* | Spain |
| III | E | *F. falciforme* | 94S | MT266939 | MT267279 | MT307310 | Emydidae: *Trachemys scripta* | Spain |
| III | E | *F. falciforme* | 91S | MT266936 | MT267280 | MT307312 | Emydidae: *Trachemys scripta* | Spain |
| III | E | *F. falciforme* | 29S | MT266940 | MT267291 | MT307323 | Emydidae: *Trachemys scripta* | Spain |
| III | E | *F. falciforme* | FRCS 2452 | JN235288 | JN235288 | JN235718 | Plumbing drains | USA |
| III | E | *F. falciforme* | NRRL22938 | DQ236380 | DQ236380 | DQ236380 | Hominidae: *Homo sapiens* | Indonesia |
| III | E | *F. falciforme* | NRRL28548 | DQ094363 | DQ236405 | DQ246889 | Hominidae: *Homo sapiens* | USA |
| III | E | *F. falciforme* | NRRL32331 | DQ094428 | DQ236470 | DQ246959 | Hominidae: *Homo sapiens* | USA |
| III | E | *F. falciforme* | NRRL28351 | DQ094358 | DQ236400 | DQ246881 | Hominidae: *Homo sapiens* | Mexico |
| III | E | *F. falciforme* | NRRL32540 | DQ094471 | DQ236513 | DQ247006 | Hominidae: *Homo sapiens* | India |
| III | E | *F. falciforme* | NRRL22781 | DQ094334 | DQ236376 | DQ246849 | Hominidae: *Homo sapiens* | Venezuela |
| III | E | *F. falciforme* | NRRL28565 | DQ094379 | DQ236421 | DQ246906 | Hominidae: *Homo sapiens* | USA |
| III | E | *F. falciforme* | NRRL32542 | DQ094473 | DQ236515 | DQ247008 | Hominidae: *Homo sapiens* | India |
| III | E | *F. falciforme* | 38S | MT266945 | MT267284 | MT307314 | Emydidae: *Trachemys scripta* | Spain |
| III | E | *F. falciforme* | 40S | MT266946 | MT267292 | MT307304 | Emydidae: *Trachemys scripta* | Spain |
| III | E | *F. falciforme* | 45A | MT266949 | MT266949 | MT307305 | Emydidae: *Trachemys scripta* | Spain |
| III | E | *F. falciforme* | NRRL43536 | EF453118 | EF453118 | EF452966 | Hominidae: *Homo sapiens* | USA |
| III | E | *F. falciforme* | NRRL25456 | DQ094344 | DQ236386 | DQ246862 | Hominidae: *Homo sapiens* | USA |
| III | E | *F. falciforme* | NRRL25746 | DQ094346 | DQ236388 | DQ246864 | Hominidae: *Homo sapiens* | USA |
| III | E | *F. falciforme* | NRRL31162 | DQ094392 | DQ236434 | DQ246919 | Hominidae: *Homo sapiens* | USA |
| III | E | *F. falciforme* | NRRL32754 | DQ094533 | DQ236575 | DQ247072 | *Turtle* | USA |
| III | E | *F. Tokinense* | FRCS2484 | JN235291 | JN235291 | JN235721 | Plumbing drains | USA |
| III | E | *F. Tokinense* | FRCS2485 | JN235294 | JN235294 | JN235724 | Plumbing drains | USA |
| III | E | *F. Tokinense* | FRCS2491 | JN235297 | JN235297 | JN235727 | Plumbing drains | USA |
| III | E | *F. Tokinense* | FRCS2542 | JN235300 | JN235300 | JN235730 | Plumbing drains | USA |
| III | *F* | *F. Tokinense* | NRRL32755 | DQ094534 | DQ236576 | DQ247073 | Plumbing drains | USA |
| III | *F* | *F. Tokinense* | FRCS2540 | JN235325 | JN235325 | JN235755 | Plumbing drains | USA |
| III | *F* | *F. Tokinense* | FRCS2541 | JN235210 | JN235210 | JN235640 | Plumbing drains | USA |
| III | *F* | *Fusarium sp.* | 23A | MT266934 | MT267275 | MT307321 | Emydidae: *Trachemys scripta* | Spain |
| III | *F* | *Fusarium sp.* | 36S | MT266942 | MT267283 | MT307313 | Emydidae: *Trachemys scripta* | Spain |
| III | *G* | *F. solani* | 93S | MT266938 | MT267278 | MT307322 | Emydidae: *Trachemys scripta* | Spain |
| III | *G* | *F. solani* | 34A | MT266941 | MT267282 | MT307311 | Emydidae: *Trachemys scripta* | Spain |
| III | *G* | *F. solani* | FRCS2445 | JN235301 | JN235301 | JN235731 | Plumbing drains | USA |
| III | *G* | *F. solani* | NRRL22779 | DQ094333 | DQ236375 | DQ246848 | Hominidae: *Homo sapiens* | New Zealand |
| III | *G* | *F. solani* | FRCS2446 | JN235302 | JN235302 | JN235732 | Plumbing drains | USA |
| III | *G* | *F. solani* | FRCS2508 | JN235303 | JN235303 | JN235733 | Plumbing drains | USA |
| III | *G* | *F. solani* | NRRL28679 | DQ094385 | DQ236427 | DQ246912 | Hominidae: *Homo sapiens* | Cuba |
| III | *G* | *F. solani* | NRRL22783 | DQ094335 | DQ236377 | DQ246851 | Phocidae: Seal | USA |
| III | *G* | *F. solani* | NRRL32492 | EU329679 | EU329679 | DQ246990 | Hominidae: *Homo sapiens* | USA |
| III | *G* | *F. solani* | NRRL32741 | DQ094522 | DQ236564 | DQ247061 | Hominidae: *Homo sapiens* | USA |
| III | *G* | *F. solani* | 42S | MT266948 | MT267286 | MT307316 | Emydidae: *Trachemys scripta* | Spain |
| III | *G* | *F. solani* | 45S | MT266950 | MT267287 | MT307317 | Emydidae: *Trachemys scripta* | Spain |
| III | *G* | *F. solani* | NRRL32810 | DQ094577 | DQ236619 | DQ247118 | Hominidae: *Homo sapiens* | USA |
| III | *G* | *F. solani* | NRRL31168 | DQ094395 | DQ236437 | DQ246922 | Hominidae: *Homo sapiens* | USA |
| III | *G* | *F. solani* | 37A | MT266943 | MT267274 | MT307303 | Emydidae: *Trachemys scripta* | Spain |
| III | *G* | *F. solani* | 37S | MT266944 | MT267273 | MT307307 | Emydidae: *Trachemys scripta* | Spain |
| III | *G* | *F. solani* | NRRL32791 | DQ094560 | DQ236602 | DQ247100 | Hominidae: *Homo sapiens* | USA |
| III | *G* | *F. solani* | FRCS2516 | JN235305 | JN235305 | JN235735 | Plumbing drains | USA |
| III | *G* | *F. solani* | NRRL32484 | DQ094449 | DQ236491 | DQ246982 | Hominidae: *Homo sapiens* | USA |
| III | *G* | *F. solani* | 42A | MT266947 | MT267285 | MT307315 | Emydidae: *Trachemys scripta* | Spain |
| III | *G* | *F. solani* | 46S | MT266951 | MT267288 | MT307318 | Emydidae: *Trachemys scripta* | Spain |
| III | *G* | *F. solani* | NRRL32737 | DQ094449 | DQ236491 | DQ246982 | Hominidae: *Homo sapiens* | USA |
| III | *G* | *F. solani* | 88S | MT266952 | MT267289 | MT307319 | Emydidae: *Trachemys scripta* | Spain |
| III | *G* | *F. solani* | NRRL25388 | DQ094341 | MH582401 | MH582421 | Hominidae: *Homo sapiens* | India |

* *Fusarium incarnatum-equiseti* type sequence (NRRL26419) and a *Fusarium oxysporum* species complex sequence (199FUS) were used in the preliminary ITS analysis.
