# Supplementary material for: Isolation and molecular identification of pathogens causing sea turtle egg fusariosis in key nesting beaches in Costa Rica
Source: PLoS One. 2025 Sep 25;20(9):e0333280. doi: 10.1371/journal.pone.0333280 (PMC12463207; doi:10.1371/journal.pone.0333280)
Supplement: S2 Table — (DOCX) [file pone.0333280.s006.docx]

**S2 Table.** Selected STEF-causing isolates collected from eggshells and nest sand for the phylogenetic analysis.

| **Isolates** | **Species name** | **Identity (%)** | **Host** | **GenBank Accession number** | | | **Source** | **Location name** |
| --- | --- | --- | --- | --- | --- | --- | --- | --- |
|  |  |  |  | ITS nrDNA | 28S nrDNA | TEF nDNA |  |  |
| C4 | *F. falciforme* | 99.70 | *Chelonia mydas* | PQ206423 | PQ206447 | PQ219647 | Eggshell | Playa Cabuyal |
| C8 | *F. falciforme* | 99.70 | *Chelonia mydas* | PQ206424 | PQ206448 | PQ219648 | Nest Sand | Playa Cabuyal |
| C13 | *F. falciforme* | 99.68 | *Chelonia mydas* | PQ206425 | PQ206449 | PQ219649 | Nest sand | Playa Cabuyal |
| G1_50A | *F. falciforme* | 99.79 | *Dermochelys coriacea* | PQ206426 | PQ206450 | PQ219650 | Eggshell | Playa Grande, Las Baulas National Park |
| G1_50B | *F. falciforme* | 99.80 | *Dermochelys coriacea* | PQ206427 | PQ206451 | PQ219651 | Eggshell | Playa Grande, Las Baulas National Park |
| G1_50C | *F. falciforme* | 99.86 | *Dermochelys coriacea* | PQ206428 | PQ206452 | PQ219652 | Eggshell | Playa Grande, Las Baulas National Park |
| G8 | *F. falciforme* | 99.79 | *Dermochelys coriacea* | PQ206429 | PQ206453 | PQ219653 | Nest sand | Playa Grande, Las Baulas National Park |
| P2C | *F. falciforme* | 99.79 | *Dermochelys coriacea* | PQ206430 | PQ206454 | PQ219654 | Eggshell | Pacuare |
| P6A | *F. falciforme* | 99.80 | *Dermochelys coriacea* | PQ206431 | PQ206455 | PQ219655 | Eggshell | Pacuare |
| P7 | *F. falciforme* | 99.79 | *Dermochelys coriacea* | PQ206432 | PQ206456 | PQ219656 | Nest sand | Pacuare |
| P10B | *F. falciforme* | 99.80 | *Dermochelys coriacea* | PQ206433 | PQ206457 | PQ219657 | Eggshell | Pacuare |
| P10C | *F. keratoplasticum* | 99.74 | *Dermochelys coriacea* | PQ206434 | PQ206458 | PQ219658 | Eggshell | Pacuare |
| P13A | *F. falciforme* | 99.79 | *Dermochelys coriacea* | PQ206435 | PQ206459 | PQ219659 | Eggshell | Pacuare |
| P22B | *F. falciforme* | 100 | *Dermochelys coriacea* | PQ206436 | PQ206460 | PQ219660 | Eggshell | Pacuare |
| P23C | *F. falciforme* | 100 | *Dermochelys coriacea* | PQ206437 | PQ206461 | PQ219661 | Eggshell | Pacuare |
| P25B | *F. keratoplasticum* | 100 | *Dermochelys coriacea* | PQ206438 | PQ206462 | PQ219662 | Eggshell | Pacuare |
| P27C | *F. keratoplasticum* | 100 | *Dermochelys coriacea* | PQ206439 | PQ206463 | PQ219663 | Eggshell | Pacuare |
| P28A | *F. keratoplasticum* | 100 | *Dermochelys coriacea* | PQ206440 | PQ206464 | PQ219664 | Eggshell | Pacuare |
| P28B | *F. keratoplasticum* | 99.85 | *Dermochelys coriacea* | PQ206441 | PQ206465 | PQ219665 | Eggshell | Pacuare |
| P33C | *F. keratoplasticum* | 99.86 | *Dermochelys coriacea* | PQ206442 | PQ206466 | PQ219666 | Eggshell | Pacuare |
| T9 | *F. falciforme* | 100 | *Chelonia mydas* | PQ206443 | PQ206467 | PQ219667 | Nest sand | Tortuguero National Park |
| T12 | *F. falciforme* | 100 | *Chelonia mydas* | PQ206444 | PQ206468 | PQ219668 | Nest sand | Tortuguero National Park |

Isolates were identified at the species level with maximum sequence similarity (percentage of identity) with GenBank sequences and then confirmed through BI and ML phylogenetic analysis of the ITS, LSU, and TEF1 gene regions.
