# Supplementary material for: Isolation and molecular identification of pathogens causing sea turtle egg fusariosis in key nesting beaches in Costa Rica
Source: PLoS One. 2025 Sep 25;20(9):e0333280. doi: 10.1371/journal.pone.0333280 (PMC12463207; doi:10.1371/journal.pone.0333280)
Supplement: S3 Table — (DOCX) [file pone.0333280.s007.docx]

**S3 Table.** FSSC members and other fungal species isolates obtained from *Chelonia mydas* and *Dermochelys coriacea* eggshells and nest sand in all study areas.

| **Isolates** | **Species name** | **Identity (%)** | **Isolation source** | **Location name** | **Common host** |
| --- | --- | --- | --- | --- | --- |
| C1* | *F. falciforme* | 99.50 | Nest sand | Playa Cabuyal | Soil fungus, Plants, animals, humans |
| C2* | *F. neocosmosporiellum* | 99.00 | Nest sand | Playa Cabuyal | Plants and animals, humans |
| C3* | *F. falciforme* | 99.00 | Nest sand | Playa Cabuyal | Soil fungus, Plants, animals, humans |
| C4B* | *F. falciforme* | 100 | Nest sand | Playa Cabuyal | Soil fungus, Plants, animals, humans |
| C5* | *F. falciforme* | 100 | Nest sand | Playa Cabuyal | Soil fungus, Plants, animals, humans |
| C6* | *F. falciforme* | 99.63 | Nest sand | Playa Cabuyal | Soil fungus, Plants, animals, humans |
| C10 | *Gymnascella hyalinospora* | 99.15 | Nest sand | Playa Cabuyal | Saprophytic, human infections |
| C12* | *F. falciforme* | 99.64 | Nest sand | Playa Cabuyal | Soil fungus, Plants, animals, humans |
| C14* | *F. neocosmosporiellum* | 99.44 | Nest sand | Playa Cabuyal | Plants and animals, humans |
| C15* | *F. neocosmosporiellum* | 99.43 | Nest sand | Playa Cabuyal | Plants and animals, humans |
| C16* | *F. falciforme* | 99.82 | Nest sand | Playa Cabuyal | Soil fungus, Plants, animals, humans |
| C17* | *F. falciforme* | 99.87 | Nest sand | Playa Cabuyal | Soil fungus, Plants, animals, humans |
| C18* | *F. neocosmosporiellum* | 99.44 | Nest sand | Playa Cabuyal | Plants and animals, humans |
| C19 | *Acrophialophora sp.* | 99.23 | Nest sand | Playa Cabuyal | Human pathogen, saprotroph |
| C20 | *Acrophialophora sp.* | 99.23 | Nest sand | Playa Cabuyal | Human pathogen, saprotroph |
| C21 | *Acrophialophora sp.* | 99.40 | Nest sand | Playa Cabuyal | Human pathogen, saprotroph |
| C22 | *Acrophialophora sp.* | 99.57 | Nest sand | Playa Cabuyal | Human pathogen, saprotroph |
| C23 | *Trichoderma longibrachiatum* | 99.67 | Nest sand | Playa Cabuyal | Saprotroph, plant pathogen |
| CB26A | *Aspergillus tamarii* | 99.00 | Eggshell | Playa Cabuyal | Plants, soil |
| CB26B | *Aspergillus flavus* | 100 | Eggshell | Playa Cabuyal | Plants, soil |
| CB26C | *Aspergillus sp.* | 100 | Eggshell | Playa Cabuyal | Plants, soil |
| CB18C | *Aspergillus tamarii* | 99.82 | Eggshell | Playa Cabuyal | Plants, soil |
| G1 | *Parengyodontium album* | 100 | Nest sand | Playa Grande, Las Baulas N.P | Soil fungus |
| G2 | *Aspergillus sydowii* | 100 | Nest sand | Playa Grande, Las Baulas N.P | Soil fungus, coral pathogen |
| G4 | *Acrophialophora sp.* | 99.18 | Nest sand | Playa Grande, Las Baulas N.P | Human pathogen, saprotroph |
| G6 | *Aspergillus terreus* | 100 | Nest sand | Playa Grande, Las Baulas N.P | Soil fungus |
| G7 | *Aspergillus terreus* | 100 | Nest sand | Playa Grande, Las Baulas N.P | Soil fungus |
| G9 | *Aspergillus terreus* | 99.82 | Nest sand | Playa Grande, Las Baulas N.P | Soil fungus |
| G10 | *Endocarpon sp.* | 99.00 | Nest sand | Playa Grande, Las Baulas N.P | Soil lichen |
| G12 | *Gymnascella hyalinospora* | 99.31 | Nest sand | Playa Grande, Las Baulas N.P | Saprophytic, human pathogen |
| G15 | *Gymnascella hyalinospora* | 99.00 | Nest sand | Playa Grande, Las Baulas N.P | Saprophytic, human pathogen |
| G16* | *F. neocosmosporiellum* | 99.43 | Nest sand | Playa Grande, Las Baulas N.P | Plants and animals, humans |
| G17* | *F. neocosmosporiellum* | 99.42 | Nest sand | Playa Grande, Las Baulas N.P | Plants and animals, humans |
| G18 | *Chaetomium globosum* | 98.88 | Nest sand | Playa Grande, Las Baulas N.P | Mycosis in humans, saprotroph |
| G4_75A | *Scedosporium aurantiacum* | 100 | Eggshell | Playa Grande, Las Baulas N.P | Human pathogen, saprotroph |
| G4_75B | *Scedosporium aurantiacum* | 100 | Eggshell | Playa Grande, Las Baulas N.P | Human pathogen, saprotroph |
| G4_75C | *Scedosporium aurantiacum* | 100 | Eggshell | Playa Grande, Las Baulas N.P | Human pathogen, saprotroph |
| G5_25A | *Aspergillus quadrilineatus* | 100 | Eggshell | Playa Grande, Las Baulas N.P | Human pathogen, soil fungus |
| G5_75A | *Gymnascella stercoraria* | 99.80 | Eggshell | Playa Grande, Las Baulas N.P | Soil saprotroph |
| G5_75B | *Thielavia sp.* | 99.12 | Eggshell | Playa Grande, Las Baulas N.P | Soil fungus, human pathogen |
| G6_100B | *Pseudallescheria ellipsoidea* | 99.82 | Eggshell | Playa Grande, Las Baulas N.P | Soil fungus, human pathogen |
| G7_25A | *Pseudallescheria ellipsoidea* | 99.79 | Eggshell | Playa Grande, Las Baulas N.P | Soil fungus, human pathogen |
| G8_75B | *Aspergillus flavus* | 100 | Eggshell | Playa Grande, Las Baulas N.P | Human pathogen, soil fungus |
| G10_502A | *Aspergillus sp* | 99.75 | Eggshell | Playa Grande, Las Baulas N.P | Soil fungus |
| P1 | *Neoscytalidium dimidiatum* | 100 | Nest sand | Pacuare | Plant and human pathogen |
| P2 | *Lasiodiplodia theobromae* | 100 | Nest sand | Pacuare | Plant pathogen |
| P3 | *Lasiodiplodia theobromae* | 100 | Nest sand | Pacuare | Plant pathogen |
| P4 | *Aspergilus hortae* | 99.79 | Nest sand | Pacuare | Soil and plants |
| P5 | *Neoscytalidium dimidiatum* | 100 | Nest sand | Pacuare | Plant and human pathogen |
| P6 | *Trichoderma longibrachiatum* | 100 | Nest sand | Pacuare | Saprotroph, plant pathogen |
| P8 | *Trichoderma longibrachiatum* | 100 | Nest sand | Pacuare | Saprotroph, plant pathogen |
| P9 | *Aspergillus terreus* | 99.81 | Nest sand | Pacuare | Soil fungus |
| P10 | *Aspergillus hortae* | 100 | Nest sand | Pacuare | Soil fungus, human pathogen |
| P11 | *Aspergillus terreus* | 99.63 | Nest sand | Pacuare | Soil fungus |
| P12 | *Aspergillus terreus* | 100 | Nest sand | Pacuare | Soil fungus |
| P13 | *Aspergillus terreus* | 100 | Nest sand | Pacuare | Soil fungus |
| P14 | *Lasiodiplodia theobromae* | 100 | Nest sand | Pacuare | Plant pathogen |
| P15 | *Trichoderma longibrachiatum* | 99.82 | Nest sand | Pacuare | Saprotroph, plant pathogen |
| P16 | *Phytopythium cucurbitacearum* | 99.51 | Nest sand | Pacuare | Crop pathogen |
| P1A* | *F. equiseti* | 100 | Eggshell | Pacuare | Saprotroph, plants, animals |
| P1B | *Coprinellus sp.* | 99.84 | Eggshell | Pacuare | Decomposer, no pathogen |
| P2A | *Mortierella sp.* | 99.67 | Eggshell | Pacuare | Saprotrophs in soil |
| P6C | *Coprinellus radians* | 100 | Eggshell | Pacuare | Decomposer, no pathogen |
| P7A | *Coprinellus sp.* | 99.84 | Eggshell | Pacuare | Decomposer, no pathogen |
| P8B | *Coprinellus radians* | 99.84 | Eggshell | Pacuare | Decomposer, no pathogen |
| P8C | *Phanerodontia chrysosporium* | 100 | Eggshell | Pacuare | Saprotroph |
| P10A* | *F. keratoplasticum* | 100 | Eggshell | Pacuare | Animal, human pathogen |
| P11A | *Hypocrea lixii* | 100 | Eggshell | Pacuare | Plant pathogen |
| P11B | *Trichoderma lentiforme* | 100 | Eggshell | Pacuare | Saprotrophs, plant pathogen |
| P11C | *Trichoderma lentiforme* | 100 | Eggshell | Pacuare | Saprotrophs, plant pathogen |
| P15A | *Coprinellus radians* | 100 | Eggshell | Pacuare | Decomposer, no pathogen |
| P16A* | *F. solani* | 100 | Eggshell | Pacuare | Human, animal pathogen |
| P16B | *Flavodon sp.* | 99.00 | Eggshell | Pacuare | Macro fungi, decomposer |
| P17B | *Phanerocaete concrescens* | 100 | Eggshell | Pacuare | Saprotroph |
| P17C | *Phanerina mellea* | 99.60 | Eggshell | Pacuare | Macro fungi, decomposer |
| P19A | *Curvularia alcornii* | 100 | Eggshell | Pacuare | Saprotroph, human infections |
| P19B | *Chaetomium sp.* | 99.00 | Eggshell | Pacuare | Human pathogen, saprotroph |
| P19C | *Chaetomium globosum* | 100 | Eggshell | Pacuare | Mycosis in humans, saprotroph |
| P20B* | *F. falciforme* | 99.00 | Eggshell | Pacuare | Soil fungus, Plants, animals, humans |
| P21A | *Phanerodontia chrysosporium* | 100 | Eggshell | Pacuare | Saprotroph |
| P21B | *Phanerodontia chrysosporium* | 100 | Eggshell | Pacuare | Saprotroph |
| P22A* | *F. falciforme* | 99.67 | Eggshell | Pacuare | Soil fungus, Plants, animals, humans |
| P22C* | *F. falciforme* | 99.69 | Eggshell | Pacuare | Soil fungus, Plants, animals, humans |
| P23A* | *F. solani* | 100 | Eggshell | Pacuare | Human, animal pathogen |
| P23B | *Coprinellus radians* | 100 | Eggshell | Pacuare | Decomposer, no pathogen |
| P24A* | *F. keratoplasticum* | 100 | Eggshell | Pacuare | Animal, human pathogen |
| P24B | *Coprinellus radians* | 100 | Eggshell | Pacuare | Decomposer, no pathogen |
| P24C | *Coprinellus radians* | 100 | Eggshell | Pacuare | Decomposer, no pathogen |
| P25A* | *F. keratoplasticum* | 100 | Eggshell | Pacuare | Animal, human pathogen |
| P25C* | *F. keratoplasticum* | 100 | Eggshell | Pacuare | Animal, human pathogen |
| P26A | *Mortierella sp.* | 99.83 | Eggshell | Pacuare | Animal, human pathogen |
| P26B | *Mortierella sp.* | 99.67 | Eggshell | Pacuare | Animal, human pathogen |
| P26C* | *F. falciforme* | 99.64 | Eggshell | Pacuare | Soil fungus, Plants, animals, humans |
| P27A* | *F. oxysporum* | 100 | Eggshell | Pacuare | Animal, human pathogen |
| P27B* | *F. oxysporum* | 100 | Eggshell | Pacuare | Animal, human pathogen |
| P28C | *Schizophyllum commune* | 100 | Eggshell | Pacuare | Macro fungi, decomposer |
| P29A | *Scedosporium boydii* | 99.82 | Eggshell | Pacuare | Plants and humans |
| P30B | *Planus strigellus* | 100 | Eggshell | Pacuare | Macro fungi, decomposer |
| P30C | *Schizophyllum commune* | 100 | Eggshell | Pacuare | Macro fungi, decomposer |
| P31B | *Trichoderma lentiforme* | 100 | Eggshell | Pacuare | Saprotrophs, plant pathogen |
| P33A* | *F. keratoplasticum* | 100 | Eggshell | Pacuare | Animal, human pathogen |
| P33B* | *F. keratoplasticum* | 100 | Eggshell | Pacuare | Animal, human pathogen |
| P34A* | *F. keratoplasticum* | 100 | Eggshell | Pacuare | Animal, human pathogen |
| P34B* | *F. keratoplasticum* | 99.83 | Eggshell | Pacuare | Animal, human pathogen |
| P34C* | *F. keratoplasticum* | 100 | Eggshell | Pacuare | Animal, human pathogen |
| T1* | *F. oxysporum* | 100 | Nest sand | Tortuguero National Park | Animal, human pathogen |
| T2 | *Trichoderma virens* | 100 | Nest sand | Tortuguero National Park | Saprotrophs, plant pathogen |
| T3 | *Trichoderma reesei* | 100 | Nest sand | Tortuguero National Park | Saprotrophs, plant pathogen |
| T4* | *F. falciforme* | 100 | Nest sand | Tortuguero National Park | Soil fungus, Plants, animals, humans |
| T5 | *Phytophytium cucurbitacearum* | 100 | Nest sand | Tortuguero National Park | Crop pathogen |
| T6 | *Trichoderma sp.* | 100 | Nest sand | Tortuguero National Park | Saprotrophs, plant pathogen |
| T8* | *F. falciforme* | 99.82 | Nest sand | Tortuguero National Park | Soil fungus, Plants, animals, humans |
| T10 | *Aspergillus terreus* | 99.78 | Nest sand | Tortuguero National Park | Soil fungus |
| T11* | *F. oxysporum* | 99.44 | Nest sand | Tortuguero National Park | Animal, human pathogen |
| T13* | *F. falciforme* | 99.82 | Nest sand | Tortuguero National Park | Soil fungus, Plants, animals, humans |
| T14* | *F. falciforme* | 99.46 | Nest sand | Tortuguero National Park | Soil fungus, Plants, animals, humans |
| T15 | *Trichoderma reesei* | 100 | Nest sand | Tortuguero National Park | Saprotrophs, plant pathogen |
| T16 | *Trichoderma reesei* | 100 | Nest sand | Tortuguero National Park | Saprotrophs, plant pathogen |
| T17* | *F. oxysporum* | 100 | Nest sand | Tortuguero National Park | Animal, human pathogen |
| T18 | *Trichoderma sp.* | 100 | Nest sand | Tortuguero National Park | Saprotrophs, plant pathogen |
| T19 | *Trichoderma sp.* | 100 | Nest sand | Tortuguero National Park | Saprotrophs, plant pathogen |
| T20 | *Trichoderma sp.* | 100 | Nest sand | Tortuguero National Park | Saprotrophs, plant pathogen |
| T21 | *Trichoderma sp.* | 100 | Nest sand | Tortuguero National Park | Saprotrophs, plant pathogen |
| T22 | *Cylindrocladiella sp.* | 100 | Nest sand | Tortuguero National Park | Plant pathogen |
| T23 | *Trichoderma sp.* | 100 | Nest sand | Tortuguero National Park | Saprotrophs, plant pathogen |

Note: Putative identity is based on the Blast searches with ITS data. Isolates are identified at the species level based on maximum sequence similarity comparisons (percentage of identity) with GenBank sequences. * Isolates identity also confirmed through BI and ML analyses of the ITS region (preliminary ITS analysis).
